# Differential Gel Electrophoresis (DIGE) Evaluation of Naphthoimidazoles Mode of Action: A Study in *Trypanosoma cruzi* Bloodstream Trypomastigotes

**DOI:** 10.1371/journal.pntd.0004951

**Published:** 2016-08-23

**Authors:** Giselle Villa Flor Brunoro, Vitor Marcel Faça, Marcelle Almeida Caminha, André Teixeira da Silva Ferreira, Monique Trugilho, Kelly Cristina Gallan de Moura, Jonas Perales, Richard Hemmi Valente, Rubem Figueiredo Sadok Menna-Barreto

**Affiliations:** 1 Laboratório de Toxinologia, Instituto Oswaldo Cruz, Fundação Oswaldo Cruz, Rio de Janeiro, Brazil; 2 Departamento de Bioquímica e Imunologia, Faculdade de Medicina de Ribeirão Preto, Universidade de São Paulo, Ribeirão Preto, Brazil; 3 Laboratório de Biologia Celular, Instituto Oswaldo Cruz, Fundação Oswaldo Cruz, Rio de Janeiro, Brazil; 4 Núcleo de Pesquisas em Produtos Naturais, Universidade Federal do Rio de Janeiro, Rio de Janeiro, Brazil; Instituto de Investigaciones Biotecnológicas, ARGENTINA

## Abstract

**Background:**

The obligate intracellular protozoan *Trypanosoma cruzi* is the causative agent of Chagas disease, a neglected illness affecting millions of people in Latin America that recently entered non-endemic countries through immigration, as a consequence of globalization. The chemotherapy for this disease is based mainly on benznidazole and nifurtimox, which are very efficient nitroderivatives against the acute stage but present limited efficacy during the chronic phase. Our group has been studying the trypanocidal effects of naturally occurring quinones and their derivatives, and naphthoimidazoles derived from β-lapachone N1, N2 and N3 were the most active. To assess the molecular mechanisms of action of these compounds, we applied proteomic techniques to analyze treated bloodstream trypomastigotes, which are the clinically relevant stage of the parasite.

**Methodology/Principal Findings:**

The approach consisted of quantification by 2D-DIGE followed by MALDI-TOF/TOF protein identification. A total of 61 differentially abundant protein spots were detected when comparing the control with each N1, N2 or N3 treatment, for 34 identified spots. Among the differentially abundant proteins were activated protein kinase C receptor, tubulin isoforms, asparagine synthetase, arginine kinase, elongation factor 2, enolase, guanine deaminase, heat shock proteins, hypothetical proteins, paraflagellar rod components, RAB GDP dissociation inhibitor, succinyl-CoA ligase, ATP synthase subunit B and methionine sulfoxide reductase.

**Conclusion/Significance:**

Our results point to different modes of action for N1, N2 and N3, which indicate a great variety of metabolic pathways involved and allow for novel perspectives on the development of trypanocidal agents.

## Author Summary

*Trypanosoma cruzi* is the etiological agent of Chagas disease, an important illness for Latin American countries that is now afflicting other continents due to the immigration of infected people. The available chemotherapy is limited to the chronic phase of the disease, being the development of novel active compounds essential, and the search for specific molecular targets for drugs in *T*. *cruzi* is necessary. In this context, our group has synthesized and screened many compounds ranging from natural to semi-synthetic naphthoquinones and derivatives on *T*. *cruzi*, displaying naphthoimidazoles N1, N2 and N3 the highest activity. Previous studies correlated phenotypic alterations by cell biology techniques as well as investigated mode of action by proteomic approaches in insect stage epimastigotes as a model. However, *T*. *cruzi* presents three morphologically distinct life stages with their own specific biological peculiarities and requirements that could be potential targets to drug intervention. Here, we evaluated the mechanism of action of N1, N2 and N3 in clinical relevant form of the parasite, bloodstream trypomastigotes, by proteomics. Our data pointed to 61 differentially abundant protein spots, being these proteins involved with cellular trafficking, protein synthesis, transduction signaling and energetic metabolism, among others, open interesting perspectives for trypanocidal strategies.

## Introduction

*Trypanosoma cruzi* is an obligate intracellular protozoan and the causative agent of Chagas disease, a neglected illness that affects millions of people in Latin America that has recently been found in non-endemic countries because of immigration related to globalization [1]. Currently, the transmission of this disease primarily depends on the ingestion of food contaminated with the feces of sucking Triatominae insects, although the classical transmission route through the vector still occurs in endemic areas [2,3]. Other routes such as blood transfusion, organ transplantation and congenital transmission can also occur [4]. This illness presents two phases (acute and chronic) that have distinct characteristics. During the acute phase, pathogenesis is associated with high parasitemia [5,6]; however, the chronic phase is divided into indeterminate and symptomatic forms, which present digestive symptoms and/or cardiomyopathy, the primary clinical manifestations [7].

The *T*. *cruzi* biological cycle involves vertebrate and invertebrate hosts and different parasite forms [8]. The infection of the mammalian host is triggered by the entry of metacyclic trypomastigotes, which invade cells and differentiate into replicative amastigotes. After the intracellular proliferation of amastigotes, they differentiate into trypomastigote, and these parasites then reach the bloodstream to infect new cells and tissues. The infection of triatomine bugs occurs during insect foraging, through the ingestion of trypomastigotes. In the insect midgut, trypomastigotes are differentiated into proliferative epimastigotes, which colonize the vector. In the triatomine´s posterior rectum, a novel differentiation occurs to form metacyclic trypomastigotes, which will then be eliminated with the insect feces, completing the life cycle when the parasite reaches the vertebrate bloodstream again [9].

At present (2016), the nitroheterocyclic agents benznidazole and nifurtimox are the only commercial drugs available for Chagas disease chemotherapy. These compounds are very efficient against acute cases, but their severe side effects and limited efficacy make their use controversial for the chronic phase. Research on the discovery of novel molecular drug targets in the parasite is justified by the high number of chronic patients without an effective treatment [10]. The preclinical active azoles posaconazole and a ravuconazole derivative named E1224 are now in clinical trials, although they have presented a high percentage of treatment failures in chronic patients, indicating that the search for alternative compounds must be continued [11].

**Fig 1:**
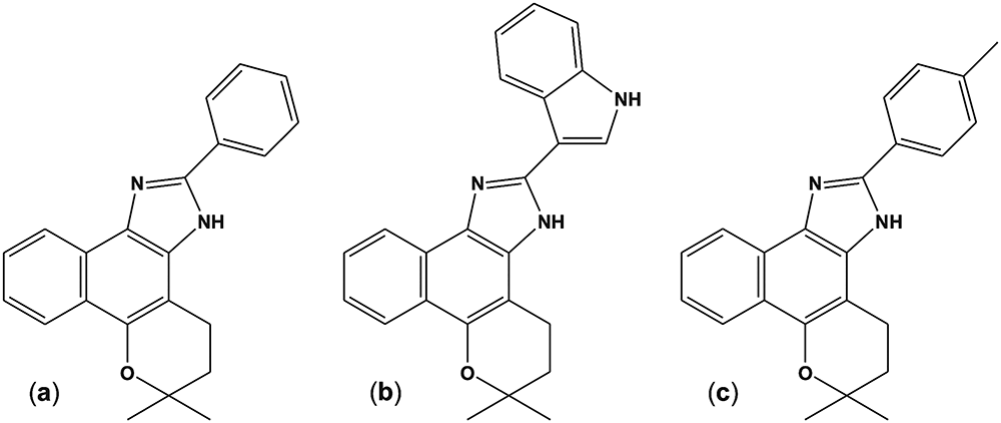
Chemical structures of β-lapachone-derived naphthoimidazoles. (a) N1, (b) N2, and (c) N3.

In searching for alternative Chagas disease chemotherapies, our group has been working on the trypanocidal effect of naturally occurring quinones, especially naphthoquinones and their derivatives, for the last 15 years. Among all the screened compounds, three naphthoimidazoles derived from β-lapachone N1, N2 and N3 (Fig 1) were the most promising [12–16]. The mechanisms of action of N1, N2 and N3 were previously assessed by cell biology techniques, and they exhibited cell cycle blockage, the inhibition of succinate cytochrome c reductase activity in epimastigotes as well as ultrastructural evidence of mitochondrial swelling, the abnormal condensation of nuclear chromatin, kinetoplast disruption and plasma membrane blebbing in bloodstream trypomastigotes. DNA fragmentation was also detected by flow cytometry and electrophoresis for the latter form of the parasite [13,14]. A cell death analysis strongly indicated autophagy as part of the naphthoimidazole mode of action, given the increase in monodansyl cadaverine labeling, the inhibition of the death process by the autophagic inhibitors wortmannin or 3-methyladenine, the overexpression of *ATG* genes and ultrastructural evidence [17].

Proteomics could play a crucial role in identifying potential drug targets because of the detection of metabolic shifts related to the pathogenesis of a great variety of diseases [18,19]. In trypanosomatids including *T*. *cruzi*, the open reading frames are organized into large polycistronic clusters that lead to post-transcriptional gene expression regulation. This molecular peculiarity supports the use of proteomic techniques as valuable technical alternatives, especially in these protozoa [20,21]. After the first description of the *T*. *cruzi* proteomic profile in 2004 [22], different high-throughput proteomic studies were performed with all stages of the parasite, and distinct strains were used to identify a maximum number of proteins from this organism [19,21,23–26].

In the chemotherapy field, only two proteomic analyses of the drug mechanism of action were performed, both of which were performed in epimastigotes. In 2008, *T*. *cruzi*'s resistance to benznidazole was assessed by using a two-dimensional gel electrophoresis (2DE) approach, which showed that the modulation of thirty-six proteins was involved in distinct metabolic pathways in resistant epimastigotes [23]. The second proteomic evaluation of trypanocidal drugs was performed by our group, and we analyzed the mechanisms of action of naphthoimidazoles N1, N2 and N3 in the *T*. *cruzi* insect stage. The most remarkable modulation was detected in mitochondrial proteins, reinforcing the electron microscopy studies [13,14]. The modulated proteins are involved in several pathways such as reactive oxygen species detoxification, protein metabolism, and structural proteins such as tubulin, among others [19]. Recently, our group assessed the proteomic map of bloodstream trypomastigotes by using a shotgun approach. *T*. *cruzi* protein entries (5,901 of them) were described in almost all the cellular compartments and metabolic pathways of the parasite, with 2,202 protein entries exclusively detected in the bloodstream forms in comparison with the culture-derived and metacyclic trypomastigote proteomic profiles reported in the literature [27], which justifies the use of bloodstream trypomastigotes as a model for pathogenesis and chemotherapy studies. In the present work, we further evaluated the mechanisms of action of the naphthoimidazoles in bloodstream trypomastigotes, which are the clinically relevant forms of the parasite, by differential gel electrophoresis (DIGE).

## Materials and Methods

### Synthesis of β-lapachone derived naphthoimidazoles

The naphthoimidazoles were obtained from a reaction of β-lapachone with aromatic aldehydes in the presence of ammonium acetate and acetic acid, leading to 4,5-dihydro-6,6-dimethyl-6H-2-(phenyl)-pyran[*b*-4,3]naphth[1,2-d]imidazole) (N1), 4,5-dihydro-6,6-dimethyl-6H-2-(3´-indolyl)-pyran[*b*-4,3]naphth[1,2-d]imidazole (N2) and 4,5-dihydro-6,6-dimethyl-6H-2-(4´-methylphenyl)-pyran[*b*-4,3]naphth[1,2-d]imidazole) (N3), as previously described [12,15,16].

### Parasites and naphthoimidazoles' treatment

*T*. *cruzi* bloodstream trypomastigotes (the Y strain) were obtained by performing a heart puncture on infected albino Swiss mice (*Mus musculus*) at peak parasitemia (7^th^ day) as previously described [27]. Six independent infections and heart punctures were performed in different days to generate six distinct pool of parasites which were considered distinct biological replicates. The parasites (5 x 10^6^ cells/mL) were treated with the three naphthoimidazoles in RPMI for 24 h at 37°C at less than a half of a dose of inhibitory concentration that led to the lysis of 50% of the trypomastigotes (IC_50_/24 h) as previously determined (10 μM of N1 and 5 μM of N2 and N3) [13,14].

### Ethics statement

In this study, the infected mice euthanized for the trypomastigotes purification in strict accordance with the recommendations of the Guide for the Care and Use of Laboratory Animals of the Brazilian National Council of Animal Experimentation (COBEA). The protocol was approved by the Committee on the Ethics of Animal Experiments of the Fundação Oswaldo Cruz (CEUA-FIOCRUZ, License Number: LW16/13).

### Protein extraction and sample preparation

After the treatment, the parasites were washed three times with phosphate-buffered saline (PBS, pH 7.4) and then incubated in sample lysis solution (7 M urea, 2 M thiourea, 4% CHAPS, 40 mM Tris, and 60 mM dithiothreitol) containing Complete Mini protease inhibitor cocktail (Roche Applied Science, Indianapolis, USA). Subsequently, 10 freezing-thawing cycles were performed and the parasite homogenate was centrifuged to separate only the soluble protein fraction as previously described [19]. The protein concentration was determined using 2D Quant kit (GE Healthcare, Buckinghamshire, England). For 2D-DIGE analysis, six independent extractions (which were considered distinct biological replicates) of the four sample types [1 control and treatments with the three naphthoimidazoles (N1, N2 and N3)] were performed.

### 2D-DIGE

In each 2D-DIGE gel, 150 μg of protein was applied, being 50 μg of control sample, 50 μg of one of the treatments from the same extraction and 50 μg of an internal standard [a pool of trypomastigotes that was made by mixing equal amounts of protein from each sample from all the extractions (45 μg of the 24 samples)]. This experimental design (S1 Table) was used to prevent the possible impairment of gel image overlays among all the samples in the DeCyder software. The sample quantity for each treatment was limited and the control sample and internal standard were present in all gels, and then the experiments were performed once as described above.

The first two sample types were alternately labeled with 400 pmol of Cy3 and Cy5, and the internal standard for all of the gels was labeled with Cy2, according to the manufacturer's protocol (GE Healthcare, Piscataway, NJ, USA). Labeled samples were applied to Immobiline DryStrips (IPG 18 cm pH 4–7) (GE Healthcare, Piscataway, NJ, USA) using an in-gel rehydration method [28]. In summary, the rehydration step was 30 V for 12h followed by voltage increment to 200 V for 1h, 500 V for 1h, 1,000 V for 1h and from 1,000 to 8,000 V in 30 min. Finally, the isoelectric focusing step was set to reach a total of 64,000 Vh at 8,000 V [29].The electric conditions for isoelectric focusing (IEF) were set to a total of 64,000 Vh at 8,000 volts. After the IEF, each strip was incubated for 15 min in 50 mM Tris-HCl pH 8.8, 6 M urea, 30% (v/v) glycerol, 2% SDS and 0.002% bromophenol blue (BPB) containing 100 mg of DTT, followed by a second 15 min incubation step with the same buffer that contained 400 mg of iodoacetamide instead of DTT. The strips were positioned on the top of a 12% T SDS-PAGE gel and overlaid with 0.5% agarose in Tris-glycine electrode buffer and 0.002% BPB. The gels were run at 2.5 W/gel for 30 min and then for a total of 100 W until the dye front reached the bottom of the gel. The first and second dimensions of gel electrophoresis were performed using IPGPhor and Ettan DaltSix systems (GE Healthcare, Piscataway, NJ, USA), respectively. The gels were then scanned with Typhoon Trio (GE Healthcare, Piscataway, NJ, USA) at a resolution of 100 μm. The photomultiplier values were adjusted to optimize sensitivity and avoid oversaturation. The resulting digitalized images were analyzed using DeCyder 5.0 software (GE HealthCare, Piscataway, NJ, USA). A differential expression analysis was performed by comparing matched spots between the treated and control groups. A paired Student´s t-test was used for a statistical analysis of the differences (p ≤ 0.01). To identify the protein spots, electrophoresis was performed on a preparative 2D-PAGE containing 400 μg of protein from the pool of all the trypomastigote samples from this study (which made up the internal standard), and the gel was stained using colloidal Coomassie blue G-250. An image overlay was performed with Image Master 2D Elite 4.01 software (GE Healthcare, Piscataway, NJ, USA).

### In-gel trypsinization

The Coomassie-stained gel spots were excised, destained with 25 mM ammonium bicarbonate pH 8.0 and 50% (v/v) acetonitrile (ACN), dehydrated with ACN and rehydrated in 15 μL of trypsin solution (20 ng/μL) for 45 min on ice (Promega, San Luis Obispo, CA, USA). A digestion was performed for 16–24 h, at 37°C. Tryptic peptides were transferred to clean tubes, and the remaining gel pieces were subjected to peptide extraction through 2 cycles involving the addition of 30 μL of 5% (v/v) formic acid/50% (v/v) ACN solution with vigorous vortexing for 20 s, resting for 15 min at room temperature, ultrasound (Ultra Cleaner 1400, Unique, Indaiatuba, SP, Brazil) for 2 min and further vortexing for 20 s. The final 80 μL was concentrated by vacuum centrifugation to approximately 10 μL and stored at -20°C until use [30]. Prior to mass spectrometry analysis, the tryptic peptides were desalted and concentrated with Zip-Tip C18 (Millipore, Billerica, MA, USA) according to the manufacturer's protocol. Peptides were eluted in 1.5 μL of 0.1% trifluoroacetic acid (TFA)/ 50% acetonitrile.

### Protein identification by MALDI TOF/TOF

The eluted peptides were mixed with an equal volume (0.3 μL) of matrix solution [10 mg/mL α-cyano-4-hydroxycinnamic acid (Sigma-Aldrich, St. Louis, MO, USA) in 50% acetonitrile, and 0.3% TFA] for analysis with a MALDI TOF/TOF 5800 Proteomics Analyzer (Applied Biosystems, Foster City, CA, USA). The instrument was operated in positive-ion delayed-extraction-reflector mode. The peptides were ionized/desorbed with 2,000 total shots per spectrum, and the spectra were acquired at a 1.77 keV accelerating potential. An external calibration was performed with the following mixture of standard peptides: Arg-bradykinin (*m*/*z* 904.468), angiotensin I (*m*/*z* 1296.685), Glu1-fibrinopeptide B (*m*/*z* 1570.677), ACTH (1–17) (*m*/*z* 2093.087) and ACTH (18–39) (*m*/*z* 2465.199) (Applied Biosystems, Foster City, CA, USA). The calibration provided a mass accuracy of 50 ppm across the mass range from 800 to 3,500 Da. Up to 10 of the most intense ion signals with signal-to-noise ratios above 30 were subjected to fragmentation, excluding three of the most common trypsin autolysis peaks (*m/z* 1045.560, *m/z* 2211.100, and *m/z* 842.500). PSD spectra were acquired using 2000 laser shots and 2.01 keV of collision energy. MS/MS spectra were calibrated using the fragment ion mass spectra of Glu1-fibrinopeptide B. All MS/MS data were analyzed using Mascot (Matrix Science, London, UK; version 2.4.1) with the following parameters: 0.40 Da of fragment ion mass tolerance and 50 ppm of parent ion tolerance; the deamidated asparagine and glutamine, oxidated methionine, carbamidomethylated cysteine and propionamide-cysteine were set as variable modifications. A search was performed against a database containing 354,024 entries [NCBInr Kinetoplastida (downloaded at May 23^rd^ 2014), *Mus musculus* (downloaded at June 16^th^ 2014) and a common contaminants database] assuming the use of trypsin as the digestion enzyme. After the search, the data were statistically validated using Scaffold 4.4.1.1 software (Proteome Software, Portland, OR, USA) [31–33]. Protein and peptides were considered identified when their Peptide Prophet-calculated probabilities were greater than 95%. Proteins without proteotypic peptide identification were grouped to satisfy the principle of maximum parsimony [34].

The identified proteins were individually categorized by subcellular localization according to the information collected from the Uniprot database (http://www.uniprot.org/) and Basic Local Alignment Search Tool of NCBI (http://blast.ncbi.nlm.nih.gov).

## Results

**Fig 2:**
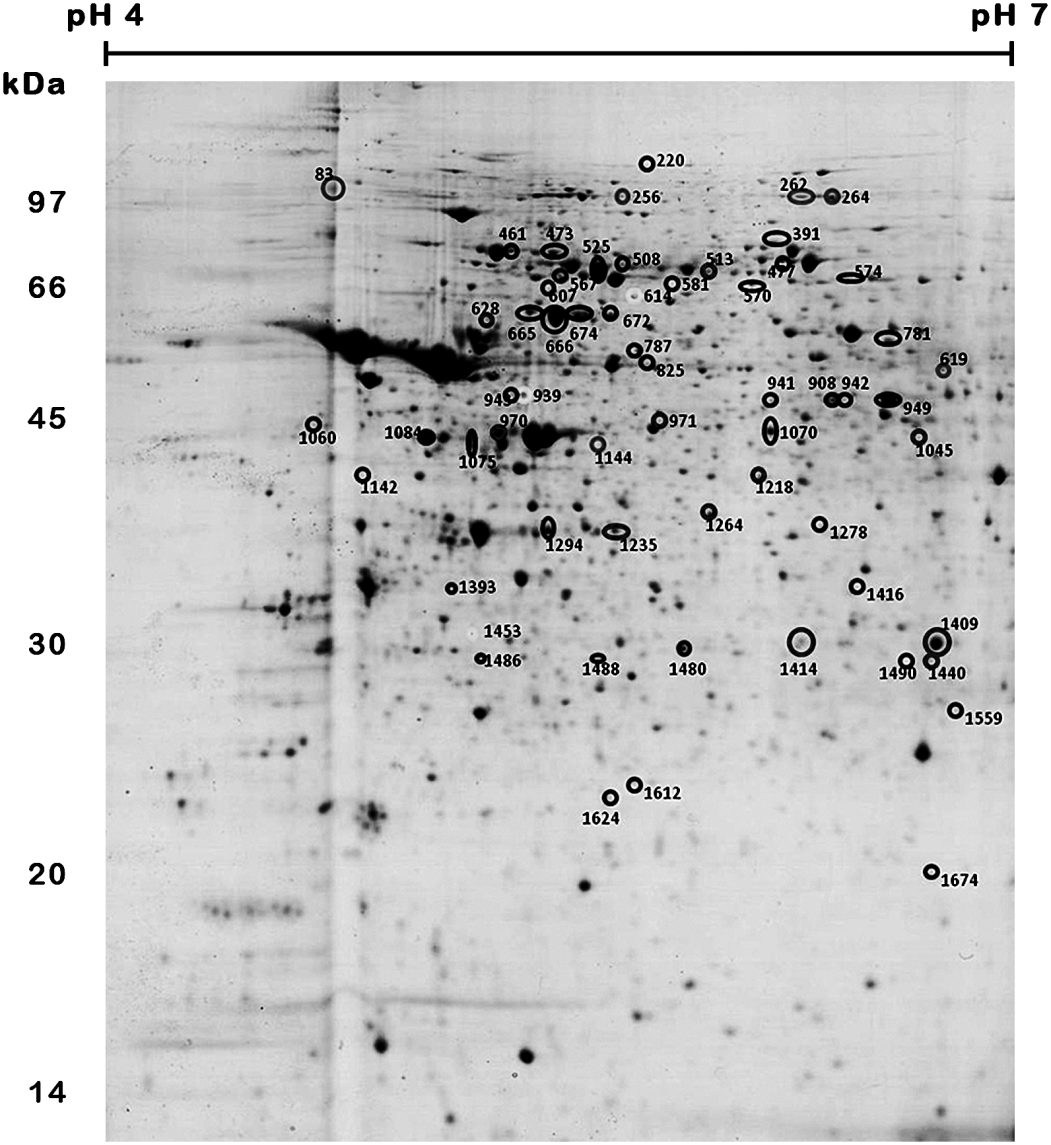
Differentially abundant protein spots from *T*. *cruzi* bloodstream trypomastigotes that were treated with naphthoimidazoles. The image depicts the preparative 2D-PAGE of a pool of all trypomastigote samples from this study that made up the internal standard. Sample (500 μg of protein) was initially separated on an 18-cm IPG strip (pH 4–7) followed by 12% SDS-PAGE. The spots that were excised from the preparative gel are indicated by the numbered circles.

**Fig 3:**
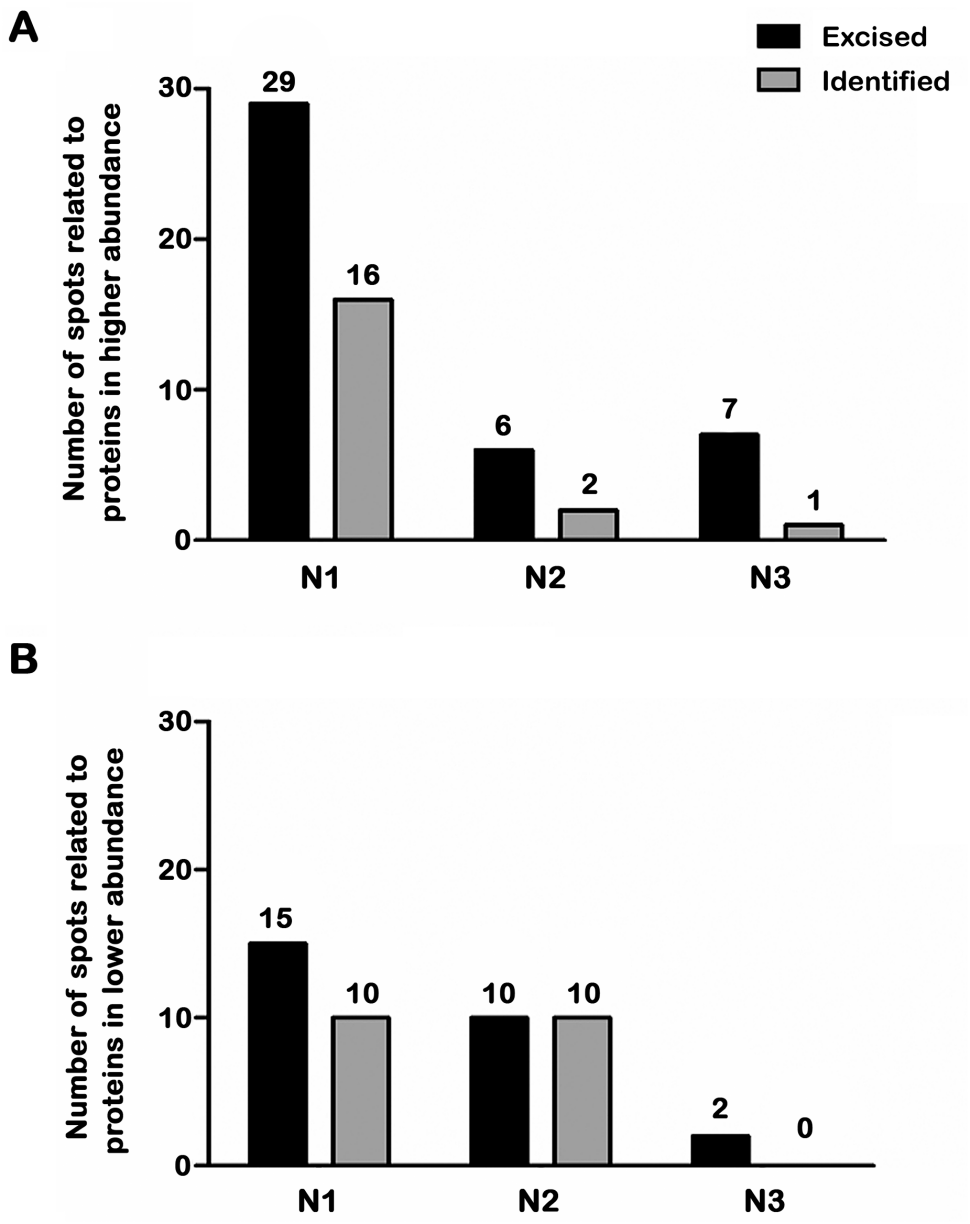
Differentially abundant protein spots from *T*. *cruzi* bloodstream trypomastigotes after naphthoimidazole treatment. Number of spots related to proteins at higher (A) and lower (B) abundance after treatment with each of the three compounds. The numbers of abundant, excised spots from the reference gel are represented by black bars and those that were effectively identified by MALDI TOF/TOF MS are shown in grey.

The treatment of bloodstream trypomastigotes with naphthoimidazoles yielded a total of 61 differentially abundant protein spots by using 2D-DIGE (Figs 2 and 3). Comparisons between the control group and N1, N2 or N3 treatments yielded 44, 16 and 9 differentially abundant protein spots, respectively (Fig 3A and 3B). The differentially abundant protein spots were not exclusive of one comparison, *e*.*g*., there are 3 common spots that are simultaneously differentially abundant in N1- and N3-treated parasites (spots 614, 939 and 1453) and 5 spots simultaneously found in N1 and N2 treatments (spots 567, 1084, 1294, 1480 and 1488) as presented in Fig 2. A total of 36, 11 and 6 spots were only found to be differentially abundant in parasites that were treated with N1, N2 and N3, respectively.

After the MALDI TOF/TOF analysis, 34 protein spots were identified by 27 distinct protein entries (Table 1 and S2 Table). Of the total, only 36 spots were found to be differentially abundant following N1 treatment, but only 21 were identified. Of these identified spots, 15 protein spots were found to be more abundant in treated samples, and 6 spots were found to be less abundant. Among the more abundant spots, the following were identified: stress response proteins [a heat shock protein at 60 kDa (spots 665, 666, and 674), a heat shock protein at 70 kDa (525) and glucose-related protein 78 (spot 461)], energy metabolism regulation proteins [vacuolar ATP synthase subunit B (spot 787), asparagine synthetase A (spot 1070) and enolase (spot 949)], nucleic acid metabolism [guanine deaminase (spot 942)], protein transport [RAB GDP dissociation inhibitor alpha (spot 941)], cell motility and structural proteins [paraflagellar rod protein (spot 473) and major paraflagellar rod protein (spot 477)], proteins involved in protein folding [chaperonin (spot 607) and hypothetical protein (spot 781). The structural protein gelsolin (spot 943) from *Mus musculus* was found to be more abundant with N1 treatment. Among the less abundant protein spots that were only detected after N1 treatment, a hypothetical protein entry (spot 1612), alpha tubulin (spot 1624), succinyl-CoA ligase (spots 1278, 1393), heat shock protein at 70 kDa (spot 1075) and peptide methionine sulfoxide reductase (spot 1674) were found. In our results, the heat shock protein at 70 kDa was identified in spots showing higher (spot 525) and lower (spot 1075) abundance after N1 treatment.

**Table 1:** Identifications of *T*. *cruzi* trypomastigotes proteins modulated by treatment with naphthoimidazoles.

Compound*	Spot numbers^§^	Protein description
↓ N1, ↓ N2	1084, 1294	85 kDa protein [*T*. *cruzi*]
↓ N2	1264	activated protein kinase C receptor, putative [*T*. *cruzi*]
↓ N1	1624	alpha tubulin [*T*. *cruzi*]
↓ N2	1440	arginine kinase, putative [*T*. *cruzi*]
↑ N1	1070	asparagine synthetase A, partial [*T*. *cruzi*]
↓ N2	1218	A-X actin [*Mus musculus*]
↓ N2	1235	beta tubulin 1.9 [*T*. *cruzi*]
↑ N1	607	chaperonin, putative [*T*. *cruzi*]
↑ N3	264	elongation factor 2 [*T*. *cruzi*]
↑ N1	949	enolase [*T*. *cruzi*]
↑ N1	943	gelsolin, isoform CRA_a [*Mus musculus*]
↑ N1	461	glucose-regulated protein 78, putative [*T*. *cruzi*]
↑ N1	942	guanine deaminase, putative [*T*. *cruzi*]
↑ N1	665, 666, 674	heat shock protein 60 kDa [*T*. *cruzi*]
↑ N1	525	heat shock protein 70 [*T*. *cruzi*]
↓ N1	1075	heat shock protein 70 [*T*. *cruzi*]
↓ N2	1409, 1414	heat shock protein 70 [*T*. *cruzi*]
↓ N1, ↓ N2	1480, 1488	heat shock protein 70 [*T*. *cruzi*]
↓ N1	1612	hypothetical protein TCDM_14147 [*T*. *cruzi*]
↑ N1, ↑ N2	567	hypothetical protein, conserved [*T*. *cruzi*]
↑ N1	781	hypothetical protein, conserved [*T*. *cruzi*]
↑ N1	477	major paraflagellar rod protein [*T*. *cruzi*]
↑ N1	473	paraflagellar rod component [*T*. *cruzi*]
↓ N1	1674	peptide methionine sulfoxide reductase [*T*. *cruzi*]
↑ N2	970	put. beta-actin (aa 27–375) [*Mus musculus*]
↑ N1	941	RAB GDP dissociation inhibitor alpha, putative [*T*. *cruzi*]
↓ N1	1278, 1393	succinyl-CoA ligase [GDP-forming] beta-chain, putative [*T*. *cruzi*]
↑ N1	787	vacuolar ATP synthase subunit B, putative [*T*. *cruzi*]

* The symbols ↑ and ↓ represent respectively higher and lower intensity of the protein spot in the treated (N1, N2 or N3) sample compared to the control.

^§^ Spot numbers are indicated in the gel image of Fig 2.

For N2 treatment, of the 11 spots that were found to be differentially abundant with this treatment, 7 were identified. Actin entries from *Mus musculus* were to be found more (spot 970) and less (spot 1218) abundant, and arginine kinase (spot 1440), beta tubulin (spot 1235), heat shock protein at 70 kDa (spots 1409 and 1414) and activated protein kinase C receptor (spot 1264) were identified as less abundant. Distinct spots, namely spot number 1624 from the N1 treatment analysis and spot number 1235 from the N2 treatment were identified as tubulin, and both were found to be less abundant in comparison with the control sample.

Evaluating the spots simultaneously uncovered those that were differentially abundant in response to N1 and N2 treatments; a hypothetical protein (spot 567) was identified as being more abundant in both treatments, and the 85 kDa protein (spots 1084 and 1294) and the heat shock protein of 70 kDa (spots 1480 and 1488) were identified as less abundant. Of the 6 spots that were found to be differentially abundant with N3 treatment, 1 was identified as elongation factor 2 (spot 264), and it was more abundant with this treatment.

**Fig 4:**
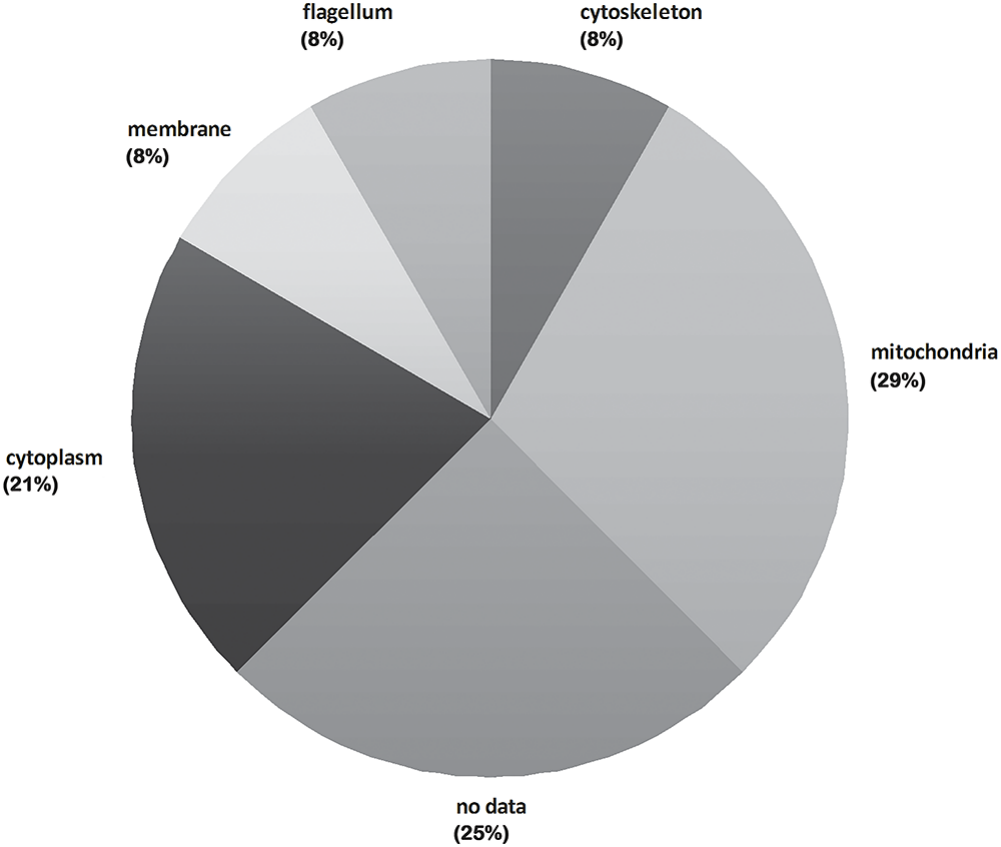
Cellular component classification of proteins that were differentially abundant in *T*. *cruzi* bloodstream trypomastigotes treated with naphthoimidazoles. The total protein number for each classification is represented as a percentage.

The subcellular localization of the 24 *T*. *cruzi* protein entries from trypanosomes is shown in Fig 4. A great number of mitochondrial proteins was identified (29%), followed by cytosolic entries (21%). Cytoskeletal, flagellar and plasma membrane proteins represent 8% each.

## Discussion

Many natural quinones that have been isolated from plant resources can participate in multiple biological oxidative processes because of their structural properties, given that their biological function is associated with their redox potential [35]. Quinoidal compounds are also sources of heterocycles, and there are few studies about the synthesis of quinone derivatives based on the reactivity of 1,2-quinoidal carbonyls towards nucleophilic reagents. Several classes of compounds were synthesized from β-lapachone, and among them, the naphthoimidazoles N1, N2 and N3 presented the highest activity against *T*. *cruzi* [12,15,16] in terms of efficacy against the three forms of the parasite [13,14].

As mentioned before, proteomics has only been employed twice in Chagas disease chemotherapy studies; for resistance/susceptibility analyses of benznidazole [23], and in a study about the mode of action of naphthoimidazoles N1, N2 and N3 [19]. In both cases, epimastigotes were used as a model, especially because of the axenic proliferation of the insect form of the parasite. The present analysis is the first proteomic study of trypanocidal drugs on *T*. *cruzi* trypomastigotes that were purified from the mammalian bloodstream, which is the most clinically relevant form. Two previous proteomic analyses of compounds against *T*. *cruzi* epimastigotes employed a two-dimensional gel approach (Coomassie blue staining) followed by MALDI-TOF/TOF identification [19,23], making it possible to obtain large amounts of samples crucial to completing all the performed analyses. However, the sample quantity represents the most important limitation during the proteomic evaluation of non-proliferative bloodstream trypomastigotes, and the need for a great number of animals makes this study very difficult to do.

Mass spectrometry-based proteomics is widely used for quantitative studies. The two main peptide-centered approaches are the label free techniques and mass-difference and isobaric tagging. The first one measure the abundance of proteins in their native state based on peptide ion intensities or spectral counts, but lack the throughput of labelling methods and the traceability of variations through analysis [36,37]. The isotopic (binary or tertiary comparisons, e.g.: ICAT, SILAC) and isobaric (multiplex analysis, e.g.: iTRAQ, TMT) labelling methods introduce respectively increased complexity in MS acquisitions and isobaric interference creating chimeric MS/MS spectra, both leading to decrease in accuracy of quantitation and limiting the sampling depth of proteome [38,39]. Withal, targeted proteomics methods (e.g., SRM and PRM) are analytically suitable experiments for quantification based on MS because of their exquisite selectivity and sensitivity [40]. However, continuing improvements in targeted approaches are currently under way, and an improved analytical workflow is still needed to ensure precise quantification [41]. The main advantage of gel-based approaches is the evaluation of the protein map (or proteoform map) of a sample regarding the hydrophobicity and molecular weight characteristics from intact polypeptides. In this context, DIGE is the state-of-the-art in two-dimensional gels because of its sensitivity, reproducibility and increased linear dynamic range for protein spot comparisons [42]. Here, the internal standard, a control sample and a treated sample of the same extraction were run in the same gel, as shown in Supplementary Table 1. As previously described [43], this experimental design was used to prevent the impairment of gel image overlays among all the samples in the DeCyder software because of the potential substantial differences among the gel images (protein patterns) that could be generated by each treatment. By having the control sample (of the corresponding extraction sample) in each gel, a comparison among the gels can also be performed as a traditional two-dimensional electrophoresis comparison, and the image overlay is guaranteed in each gel (control and treatment). No substantial differences among the gel images were observed, and the overall image overlay was successfully performed using DeCyder software.

The DIGE approach was clearly decisive for assessing the quantitative proteomic map of bloodstream trypomastigotes that were treated with naphthoimidazoles. This assessment is better evidenced by the reduction in the sample amount in comparison with our previous epimastigote work. In that study, 500 μg of each sample was added per gel [19], and it was reduced 10-fold (50 μg/sample) in our experimental design here. The fluorescence approach led to the detection of 1,724 protein spots (three-fold more than those found by Coomassie blue staining technique for the epimastigotes) in the narrower pH 4–7 gradient applied here. Interestingly, despite the increase in the number of detected protein spots, the number of differential protein spots that was identified was quite similar, at 30 and 34 for epimastigotes and trypomastigotes, respectively.

As with epimastigotes, the choice of the concentration for the three compounds was based on previously calculated IC_50_/24 h values [13,14], never exceeding half the dose that induces lysis in 50% of the parasites, to prevent undesirable and non-specific effects. Our previous proteomic study indicated that the most remarkable number of modulated proteins in epimastigotes were mitochondrial [19]. Here, we showed that a great number of mitochondrial proteins (7 of 27 differentially abundant proteins) were present at altered levels in treated trypomastigotes. These data were reinforced by ultrastructural and biochemical evidence showing that this organelle was the primary target of naphthoimidazoles [13,14]. Unlike what was described for treated epimastigotes, none of the differentially abundant proteins identified in trypomastigotes treated with these compounds was detected in benznidazole-resistant epimastigotes by Andrade and co-workers (2008), likely because a different parasite form was used.

Among the trypomastigote proteins that were differentially abundant after the treatment, chaperones were the most recurrent proteins that were modulated. Heat shock proteins 60, 70 and 85 (spots 461, 525, 607, 665, 666, 674, 1075, 1084, 1294, 1409, 1414, 1480, and 1488) were modulated by N1 and N2, as observed in epimastigotes that were treated with the same naphthoimidazoles [19]. Interestingly, an overexpression of heat shock proteins 60 and 70 was detected in both trypomastigotes and epimastigotes after N1 treatment, suggesting a parasite injury derived from proteolytic and/or oxidative stress that led to an increase in the chaperone content. However, a decrease in the chaperone levels was observed, especially with regards to the heat shock protein 85 content, in N1- and N2-treated bloodstream and insect forms; this finding deserves further functional analysis. This reduction could be attributed to induced oxidative dysfunction, as described for other cell models [44].

After treatment with N1, N2 and N3, tubulin was the most down-regulated protein in epimastigotes as previously observed. ELISA assays indicated a decrease in the tyrosinated tubulin content, but the levels of acetylated tubulin were not altered in treated epimastigotes [19]. The tyrosinated isoform is directly related to labile microtubules that participate in vesicular trafficking and also make up the intranuclear mitotic spindle, and detyrosination has been shown to act as an important checkpoint in the trypanosomatid cell cycle [45]. The acetylated isoform is present in stable microtubules that were localized in the flagella and in the subpellicular cage of the parasite [46]. Our present data showed that tubulin was also down-regulated in trypomastigotes that were treated with N1 and N2 (spots 1235 and 1624). As observed in treated epimastigotes, no ultrastructural injury was detected in the subpellicular and flagellar microtubules of trypomastigotes after naphthoimidazole treatment [13,14], reinforcing the hypothesis that a reduction in the tyrosinated tubulin levels could also compromise vesicular trafficking in the bloodstream forms.

Here, elongation factor 2 (spot 264) was up-regulated in N3-treated trypomastigotes. In epimastigotes, this elongation factor as well as elongation factors 1-alpha and 1-beta were down-regulated by N1 and N2 [19]. These GTP-dependent enzymes participate in protein synthesis in eukaryotes [47], suggesting an impairment of the *T*. *cruzi* protein synthesis machinery, but complementary experiments must be performed to confirm the hypothesis.

An activated protein kinase C receptor (spot 1264) was down-modulated by N2, as found before in N3-treated epimastigotes [19], indicating that signaling cascades could participate in the mode of action of these compounds. The regulation of several transporter systems was performed by protein kinase C, which was also implicated in the host cell infection [48]. A decrease in protein kinase C receptor expression could lead to a consequent reduction in *T*. *cruzi* transduction signaling, impacting the parasite's infectivity.

Enolase (spot 949) was up-modulated by N1 treatment, similar to that observed in N3-treated epimastigotes [19]. This enzyme is responsible for an essential step in energetic metabolism, which is the reversible conversion of 2-phosphoglycerate to phosphoenolpyruvate in glycolysis and gluconeogenesis [49]. The increase in this enzyme level can represent a mechanism to compensate for the energetic misbalance caused by these drugs.

Furthermore, some proteins were differentially abundant in bloodstream forms that were treated with naphthoimidazoles, but these differences were not detected in epimastigotes after the treatment. This result is expected because of the significant variation between the proteomic map of both parasite forms [24]. Some enzymes from distinct metabolic pathways were up-regulated in N1-treated parasites. Asparagine synthetase A (spot 1070) is responsible for the production of asparagine from aspartate, a non-essential amino acid that is present in several proteins. Recently, an *in vitro* study suggested one inhibitor of this enzyme as an alternative target in African trypanosomiasis [50]. One possible hypothesis is that N1 treatment may reduce the content of asparagine-containing proteins, and, as a result, the level of asparagine synthetase is increased, but assays with this labeled amino acid should be performed for confirmation.

ATP synthase subunit B (spot 787) is directly related to energetic metabolism by using the ions that flux to ATP synthesis in the mitochondrion [51]. The succinyl-CoA ligase [GDP-forming] beta-chain (spots 1278, 1393) catalyzes the reversible reaction of succinyl-CoA to succinate in the citric acid cycle [52]. The extensive mitochondrial swelling and the decrease in this organelle membrane potential was previously observed [13,14], together with the modulation of mitochondrial proteins described here, which strongly indicated that the essential pathways in *T*. *cruzi* mitochondrion are a primary target of N1, and the likely energetic failure that ensues leads to parasite death.

However, peptide methionine sulfoxide reductase (spot 1674) was down-regulated in trypomastigotes after N1 treatment. This enzyme reduces methionine sulfoxide to methionine, protecting the cells from oxidative damage. In *T*. *cruzi*, antioxidant studies focused especially on the trypanothione system, given that the mechanisms for repairing oxidized proteins are very poorly described [53]. A reduction in the methionine sulfoxide reductase in treated parasites leads to an accumulation of non-repaired oxidized macromolecules in the protozoa. Interestingly, trypanothione synthetase was up-regulated in epimastigotes after treatment with three naphthoimidazoles; however, this enzyme was not differentially abundant in treated bloodstream trypomastigotes. Despite the absence of redox properties in these compounds that could lead to reactive oxygen species generation, unpublished data from our group pointed to the reversion of the trypanocidal effect of N1, N2 and N3 by classical antioxidants such as tocopherol and urate in both epimastigotes and trypomastigotes. Pre-incubation with these antioxidant agents also led to a decrease in the reactive species produced by naphthoimidazole treatment in the insect forms (Menna-Barreto, personal communication). Moreover, the up-regulation of guanine deaminase (spot 942) after N1 treatment was observed. This enzyme converts guanine to xanthine in purine metabolism. Subsequently, the reaction of xanthine with water and molecular oxygen, as catalyzed by xanthine oxidase, produces urate and hydrogen peroxide [54]. This reaction could indirectly explain the reactive oxygen species produced by these naphthoimidazoles. However, a complementary analysis that employs different biochemical and molecular techniques will be performed to elucidate the mechanisms that are involved.

Some cytoskeleton-associated proteins were up-regulated in N1-treated parasites as follows: RAB GDP dissociation inhibitor alpha (spot 941) and two different components of the paraflagellar rod (spots 473 and 477). This inhibitor prevents the Rabs function in the docking step during vesicular trafficking through the inhibition of the GDP dissociation and the subsequent GDP/GTP exchange [55]. This finding suggests an impairment of cellular trafficking that is reinforced by the reduction in tubulin levels as discussed above. However, the paraflagellar rod is an extra-axonemal structure typical of trypanosomatids that are made of several proteins, and it plays a pivotal role in flagellum beating [56]. The overexpression of some elements of this structure could represent a compensatory mechanism for the misassembly of some flagellar accessory proteins that culminates in a reduction of parasite motility as previously detected [13,14].

Interestingly, one protein was down-regulated only in N2-treated trypomastigotes. Arginine kinase (spot 1440) is the transferase that is responsible for the production of phospho-L-arginine from L-arginine during an ATP-dependent reaction. Phospho-L-arginine was found to represent the energetic reservoir, and it is crucial for epimastigote proliferation [57]. Arginine kinase activity has been considered as a regulator of this process, and the decrease in this enzyme level could lead to the energetic collapse of the parasite.

In 2005, El-Sayed and colleagues sequenced the complete genome of the *T*. *cruzi* CL-Brener strain, but a huge quantity of the genes were annotated as hypothetical genes because of their absence of any putative biological functions [58]. Three different hypothetical proteins were identified in trypomastigotes that were treated with N1 (spots 567, 781 and 1612) and N2 (spot 567). A BLAST analysis showed 38% shared identity and 76% coverage (E-value: 5e^-110^) of the hypothetical protein entry of spot 567 with a putative mitochondrial nucleolar protein (gi|928109699), and 99% shared identity and 86% coverage (E-value: 0.0) for the entry of spot 781 with hydroxymethylglutaryl-CoA synthase (gi|686631215). The predicted sequence of spot 1612 did not show similarities with any protein family.

Ultimately, mammalian gelsolin and actin (spots 943, 970 and 1218) were also identified as modulated proteins after the treatment. In our recent description of the proteomic map of bloodstream trypomastigotes, we discussed the presence of blood components, especially the plasma, erythrocytes and platelets of *Mus musculus* in these samples [27]. Even the high stringency of the parasite purification process is not enough to avoid the identification of host proteins. The adsorption or the specific binding of the mammalian proteins on the parasite surface could not be discarded.

Our data together with a previous analysis indicate that the mechanisms of action of the three naphthoimidazoles are complex, and they involve distinct metabolic pathways such as cellular trafficking, protein synthesis, transduction signaling and energetic metabolism, among others, open interesting perspectives for trypanocidal strategies. Further studies on these metabolic interactions are necessary to answer some outstanding questions. Even so, new strategies for drug design have been improved by recent outcomes in *T*. *cruzi* biochemistry, allowing for better comprehension of the effects of trypanocidal agents.

The accession numbers for proteins mentioned in the text are listed as follows: elongation factor 2 [*T*. *cruzi*] gi|407835084; glucose-regulated protein 78, putative [*T*. *cruzi*] gi|407842744; paraflagellar rod component [*T*. *cruzi*] gi|2209137; major paraflagellar rod protein [*T*. *cruzi*] gi|162179; heat shock protein 70 [*T*. *cruzi*] gi|205278868; hypothetical protein, conserved [*T*. *cruzi*] gi|70871170; chaperonin, putative [*T*. *cruzi*] gi|70885659; heat shock protein 60 kDa [*T*. *cruzi*] gi|1495230; heat shock protein 60 kDa [*T*. *cruzi*] gi|1495230; heat shock protein 60 kDa [*T*. *cruzi*] gi|1495230; hypothetical protein, conserved [*T*. *cruzi*] gi|70883145; vacuolar ATP synthase subunit B, putative [*T*. *cruzi*] gi|70870795; RAB GDP dissociation inhibitor alpha, putative [*T*. *cruzi* marinkellei] gi|407410583; guanine deaminase, putative [*T*. *cruzi*] gi|70874663; gelsolin, isoform CRA_a [*Mus musculus*] gi|148676699; enolase [*T*. *cruzi*] gi|407849788; cytoplasmic beta-actin, partial [*Mus musculus*] gi|49868; asparagine synthetase A, partial [*T*. *cruzi*] gi|348658746; heat shock protein 70 [*T*. *cruzi*] gi|205278868; 85 kDa protein [*T*. *cruzi*] gi|162111; A-X actin [*Mus musculus*] gi|309090; beta tubulin 1.9 [*T*. *cruzi*] gi|18568139; activated protein kinase C receptor, putative [*T*. *cruzi*] gi|70882943; succinyl-CoA ligase [GDP-forming] beta-chain, putative [*T*. *cruzi*] gi|407849036; 85 kDa protein [*T*. *cruzi*] gi|162111; succinyl-CoA ligase [GDP-forming] beta-chain, putative [*T*. *cruzi*] gi|407849036; heat shock protein 70 [*T*. *cruzi*] gi|205278868; 70 kDa heat shock protein [*Trypanosoma rangeli*] gi|119394469; arginine kinase, putative [*T*. *cruzi*] gi|407844351; heat shock protein 70 [*T*. *cruzi*] gi|205278868; heat shock 70 kDa protein, putative [*T*. *cruzi*] gi|70876223; hypothetical protein TCDM_14147 [*T*. *cruzi* Dm28c] gi|557861434; alpha tubulin [*T*. *cruzi*] gi|1220545; peptide methionine sulfoxide reductase [*T*. *cruzi* strain CL Brener] gi|71405176.

## Supporting Information

S1 Table Experimental design for CyDye labelling in each sample and gel map. (XLSX)

S2 Table Proteins identified from DIGE spots of T. cruzi bloodstream trypomatigotes by MALDI-TOF/TOF MS. (XLSX)
